# Does the radiofrequency impedance-controlled endometrial ablation have any morphologic effects on uterine leiomyomata?: Report of 3 cases

**DOI:** 10.1186/1746-1596-3-28

**Published:** 2008-07-01

**Authors:** Oluwole Fadare, Sa A Wang, Idris L Renshaw

**Affiliations:** 1 Department of Pathology, Wilford Hall Medical Center, Lackland Air Force Base, San Antonio, TX, USA; 2 Department of Pathology, University of Texas Health Science Center at San Antonio, San Antonio, TX, USA; 3 Department of Pathology, University of Massachusetts Medical Center, Worcester, MA, USA; 4 Vanguard Pathology Associates, Austin, TX, USA

## Abstract

A variety of novel endometrial ablation technologies are now in routine use. A subset of uteri that had previously undergone these treatments will ultimately be evaluated by the pathologist. However, the full spectrum of histologic changes that may result from these treatments has received only sporadic attention. The NovaSure™ [Hologic Corporation, Marlborough, MA, USA] endometrial ablation system is one of several available second-generation technologies and its particular endometrial ablative power is based on the delivery of radiofrequency energy. The present analysis was designed to decipher any histologic changes (if any) associated with the NovaSure™ endometrial ablation system relative to benign smooth muscle tumors of the uterine corpus. Over a one-year period, 3 uteri that had previously undergone the NovaSure™ endometrial ablation and which also had leiomyomatous mass lesions were evaluated. The leiomyomatous mass lesions were extensively sampled and were evaluated for cellular shapes (epithelioid change, cellular rounding, extraordinary cytoplasmic eosinophilia, clear cell change, cytoplasmic vacuolation), nuclear changes (nucleomegaly, nucleolomegaly, multinucleation, hyperchromasia, symplastic changes), necrosis (coagulative and/or infarct), mitotic activity, apoptotic bodies or pyknotic cells, myxoid change, hyalinization. The three uteri were resected 61, 47 and 74 (mean 60.7) days post-ablation. After a detailed evaluation of multiple submucosal, intramural and subserosal leiomyomata from these 3 uteri, no noteworthy histologic changes were identified in the tumors. Since the presence or absence of tumor necrosis is one histologic criterion by which malignant potential is assigned to uterine smooth muscle neoplasms, defining any extrinsic processes that may establish, or contribute to this finding is clinically relevant. The findings reported herein suggests that if a leiomyoma that was obtained from a patient that had recently undergone the NovaSure™ endometrial ablation displays any degenerative changes such as necrosis, the changes are probably not attributable to the ablation.

## Introduction

Endometrial ablation entails the destruction of the endometrial lining by one of several energy forms that is delivered through a hysteroscope-like or similar instrument. Some of the available endometrial ablation technologies include The NovaSure™ system [Hologic (Cytyc) Corporation, Marlborough, MA, USA, which ablates the endometrial surface via radiofrequency energy], the ThermaChoice UBT (Gynecare Inc, Somerville, NJ, USA) and Cavaterm (Wallsten Medical, Morges, Switzerland) systems (which deliver thermal energy from heated fluid in a balloon), the HerOption system [American Medical Systems Inc, Minnetonka, MN, USA, which is based on freezing the endometrium surface], the HTA system [BEI Medical/Boston Scientific, Natick, MA, USA, which is based on the use of heated saline], and the Microsulis system [Microsulis Medical Ltd, Pampano Beach, FL, USA, which is based on microwave energy].

As noted previously, NovaSure™ is one of several second-generation endometrial ablation systems and has been used with increasing frequency over the past decade. The NovaSure™ system received approval from the United States Food and Drug Administration (FDA) in 2001 for the permanent ablation of the endometrial lining of women with menorrhagia that can be attributed to non-neoplastic causes [1,2]. The endometrial ablative power is derived from radiofrequency energy. Briefly, a conformable bipolar electrode array that is mounted on an expandable frame is transcervically inserted into the endometrial cavity. The array expands to form a confluent lesion on the entire internal surface of the endometrium. Radiofrequency energy is then transmitted for a period of approximately 90 seconds, and the endometrium and the superficial myometrium are thereby ablated. Increasing tissue depth of ablation causes an automatic cessation of power-delivery at a threshold of 50 ohms or at 120 seconds, and the maximum power requirements are predetermined [1,2] Recently, we encountered a distinctive uterus that was removed from a patient who had undergone the NovaSure™ endometrial ablation *and *received a gonadotropin-releasing hormone receptor agonist (Leuprolide acetate) within the 7 months that preceded her hysterectomy. The leiomyomata in the sample displayed extensive infarct-type degeneration, which we attributed to the Leuprolide acetate pretreatment. However, the case raised the possibility that the NovaSure™ endometrial ablation may have contributed to the observed changes. The present analysis was designed to decipher any histologic changes (if any) associated with the NovaSure™ endometrial ablation system relative to benign smooth muscle tumors of the uterine corpus, since degenerative changes such as necrosis are components of the diagnostic criteria by which pathologists assign malignant potential to these tumors.

## Case presentations

Three uteri that had previously undergone the NovaSure™ endometrial ablation and which also had leiomyomatous mass lesions were evaluated in detail to determine if this treatment is associated with any morphologically recognizable changes. The 3 cases represented all such cases evaluated over a one-year period. The first and second patients, a 49-year-old African-american (case 1) and a 45-year old caucasian (case 2), both presented with long-term menorrhagia that was refractory to a variety of treatments. They underwent the NovaSure™ endometrial ablation without complications. Sixty-one (case 1) and 47 (case 2) days later, they each underwent a simple hysterectomy for persistent menorrhagia. The third patient, a 51-year-old caucasian (case 3), underwent the ablation procedure for persistent menometrorrhagia, and similarly underwent a simple hysterectomy for persistent bleeding 74 days after the ablation. None of the 3 patients received hormonal treatments for their uterine masses. The cut surfaces of all the leiomyomata in all 3 cases displayed their typical whorled appearance and none displayed any evidence of degeneration or hemorrhage. In the case 1 uterus, there was a solitary, 7.8 cm intramural mass in the posterior wall of the corpus. This mass was 0.9 cm from the endometrial surface at the closest point. Twenty-five large sections were processed from this mass. In the case 2 uterus, there were a total of 4 corporal masses, including 1 submucosal (1.4 cm), intramural (5.7 cm), and 2 subserosal (1.1 cm, 2.4 cm). A total of 35 large sections were processed from these masses. In the case 3 uterus, there were 2 posterior wall masses in the corpus, one of which was submucosal and which measured 3.7 cm in maximum dimension, and the other of which was intramural and which measured 4.6 cm in maximum dimension. A total of 25 large sections were processed from these masses. All sections were then evaluated in detail for any morphologic changes that were notably distinct from what would typically be expected in a leiomyoma. Each case was evaluated for cellular shapes (epithelioid change, cellular rounding, extraordinary cytoplasmic eosinophilia, clear cell change, cytoplasmic vacuolation), nuclear changes (nucleomegaly, nucleolomegaly, multinucleation, hyperchromasia, symplastic changes), necrosis (coagulative and/or infarct), mitotic activity, apoptotic bodies or pyknotic cells, myxoid change, hyalinization etc.

**Figure 1:**
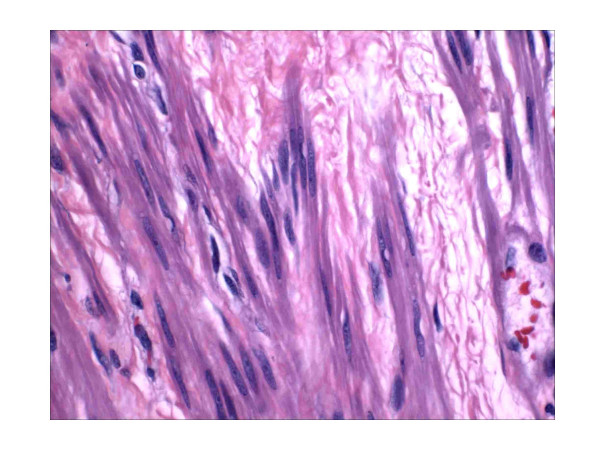
**A submucosal leiomyoma from case 2 displaying typical cytologic features.**(hematoxylin and eosin, 180×).

All cases showed typical morphologic features of benign smooth muscle neoplasms irrespective of whether the section was from a submucosal, intramural or subserosal mass (figure 1). Notably, none of the leiomyomata displayed any necrosis or indeed any evidence of previous or ongoing degeneration. The average mitotic index for the 3 cases was 0.4 per 50 high power fields.

## Discussion

Prospective studies with long-term follow-up have shown that the NovaSure™ endometrial ablation system is an efficacious modality to manage menorrhagia, with very low complication rates and high success rates regarding stoppage of excessive bleeding [3-5]. Nevertheless, in 2.9 to 8% of cases, surgical re-intervention is necessary due to treatment failures (persistent bleeding), complications, or other unrelated indications that may arise after the ablation [3-6]. The resultant uteri thereby come to pathologic attention and may show distinctive morphologic changes [7]. We previously reported the pathologic findings of one uteri that was removed 38 days after the ablation. These included a distinctive 3- to 6-mm-thick, hyalinized, subendometrial band, severe endometrial stromal fibrosis, some endometrial myxoid change, and a lack of significant changes in adenomyotic aggregates and leiomyomata in the myometrium beneath the band [7]. Livengood et al reported the histologic changes associated with "treatment failures" of a variety of endometrial ablation technologies, NovaSure™ included [8] The authors found that for water-balloon and radiofrequency based modalities, persistent post-ablation bleeding is related to "treatment-related thermally-fixed vessels" [8]. There are no other published studies specifically centered on the histologic changes associated with ablation technologies to the author's knowledge. The importance of defining the histologic spectrum brought about by these modalities lies not only in the better understanding of changes associated with failures of these treatments, but in not misclassifying an iatrogenic change as a biologic and/or neoplastic one.

In the present analysis, the authors segregated three leiomyomata-laden uteri that had previously undergone the NovaSure™ endometrial ablation over a one-year period, to determine if this treatment is associated with any morphologically recognizable changes in the smooth muscle tumors. After a detailed evaluation of such neoplasms, no distinctive histologic changes were identified.

It is concluded that the NovaSure™ endometrial ablation probably does not cause any morphologically recognizable changes to leiomyomata being evaluated at an average of 60.7 days after treatment. It is unclear if tumors removed within a shorter time-frame post-ablation will display any changes. However, hysterectomies are typically performed within the time frames noted herein, when sufficient time has passed post-ablation for all parties to be convinced of its ineffectiveness for that particular patient, and for a surgical procedure to be scheduled. Since the presence or absence of tumor necrosis is one histologic criteria by which malignant potential is assigned to uterine smooth muscle neoplasms, defining any extrinsic processes that may establish, or contribute to this finding is clinically relevant. The findings reported herein suggests that if a leiomyoma that was obtained from a patient that had recently undergone the NovaSure™ endometrial ablation displays any degenerative changes such as necrosis, the changes are probably not attributable to the ablation.

## Conflict of interests

The authors declare that they have no competing interests.

## Authors' contributions

OF, SAW and ILR co-wrote the manuscript.
